# Female infertility, Alexithymia and Stress

**DOI:** 10.1192/j.eurpsy.2023.1348

**Published:** 2023-07-19

**Authors:** A. M. Delgado Campos, P. Alcindor Huelva, A. Alvarez Astorga, S. Rubio Corgo, E. Pérez Vicente, M. Arrieta Pey, C. Diaz Gordillo, P. del Sol Calderón, A. C. Martín Martín

**Affiliations:** Departamento Psiquiatría CSM, Hospital Universitario Infanta Leonor, Madrid, Spain

## Abstract

**Introduction:**

In this research the Paris School (I.P.S.O.), by P. Marty, is chosen as the theoretical and clinical basis of Psychosomatics. We work with the degree of mentalization (good, bad and uncertain) -obtained through Marty’s Psychosomatic Classification-, as a previous diagnosis and prognosis of 120 infertile women undergoing treatment at the Assisted Reproduction Unit (ARU) at Hospital Universitario 12 de Octubre in Madrid.

**Objectives:**

a) To analyse the statistical coincidence between female infertility, stress and alexithymia syndrome. b) To verify the differences between psychosomatic disorders and other somatoform symptoms and syndromes (conversive and hypochondriac). c) To test the following hypothesis: subjects whose degree of mentalization is deficient, present high degree of alexithymia and stress.

**Methods:**

120 infertile women undergoing treatment with Assisted Reproduction Techniques were examined by means of psychodiagnostic tests.

Diagnostic tools: P. Mary’s Psychosomatic Classification (P.C.) (semi-structured interview), as a means of diagnosing the degree of mentalization; T.A.S. (Toronto Alexithymia Scale); Battery of stress measurement questionnaires (H.A.D., PANAS. IRE, MCMQ).

The correlation of coincidence between the results of C.P. and the different Alexithymia and Stress questionnaires with the independent variable (success or failure of pregnancy in the selected subjects) has been studied, applying Spearman’s Correlation Coefficient.

**Results:**

With respect to what was obtained in the Psychosomatic Classification:

- T.A.S. questionnaire yields a coefficient of [-0.48]. Therefore, there is a negative correlation between the degree of mentalization and the presence of alexithymia; in other words, as the degree of mentalization increases, the degree of alexithymia decreases and vice versa;

- There is positive correlation [0.39] between the results of Mentalization (Psychosomatic Classification) and the degree of stress; therefore, the existence of stress does not prevent better mentalization.

**Conclusions:**

The present research concludes: a) that people at risk for psychosomatic disorders have high scores on “alexithymia”; b) that patients at high risk for psychosomatic disorders do not necessarily suffer from “stress” situations; c) that there are many indicators in behavior and psychological functioning that differentiate psychosomatic disorders from conversive and hypochondriac disorders - both in their etiology and their development; d) there is a statistical correlation between female infertility and alexithymia; e) there is no statistical correlation between female infertility and stress.

**Disclosure of Interest:**

None Declared

